# Oncoviruses and melanomas: A retrospective study and literature review

**DOI:** 10.1002/jmv.27924

**Published:** 2022-06-20

**Authors:** Giulia Ciccarese, Francesco Drago, Francesco Broccolo, Angela Pastorino, Laura Pizzatti, Laura Atzori, Luca Pilloni, Davide Santinelli, Alice Urbani, Aurora Parodi, Carlo Tomasini, Franco Rongioletti

**Affiliations:** ^1^ Dermatology Unit, IRCCS Ospedale Policlinico San Martino Genoa Italy; ^2^ Department of Medicine and Surgery, School of Medicine University of Milano‐Bicocca Monza Italy; ^3^ Laboratory Cerba HealthCare Milano Italy; ^4^ Pathology Unit, Galliera Hospitals Genoa Italy; ^5^ Dermatology Unit, Department of Medical Sciences and Public Health University of Cagliari Cagliari Italy; ^6^ Pathology Service, Department of Medical Sciences and Public Health University of Cagliari Cagliari Italy; ^7^ Division of General Medicine Asl 3 Genovese Genoa Italy; ^8^ Dermatology Clinic, Fondazione IRCCS Policlinico San Matteo Pavia Italy; ^9^ Department of Clinical‐Surgical, Diagnostic and Pediatric Sciences University of Pavia Pavia Italy; ^10^ Dermatology Clinic Vita‐Salute S.Raffaele University Milan Italy

**Keywords:** cutaneous melanoma, Epstein Barr virus (EBV), human papillomavirus (HPV), mucosal melanoma, ocular melanoma, oncoviruses

## Abstract

The role of human oncoviruses in melanoma has been poorly investigated. The aim of this study was to investigate the association between oncoviruses and melanomas searching for human papillomavirus (HPV), Epstein Barr virus (EBV), and human herpesvirus 8DNA in melanoma specimens. Formalin‐fixed and paraffin‐embedded tissue specimens of cutaneous, mucosal, and ocular melanomas (OM) were selected from the Pathology Departments of the Galliera Hospital (Genoa) and the University Hospitals of Turin and Cagliari. Cutaneous and mucosal nevi have been collected as controls. The oncoviruses search has been performed with different polymerase chain reaction reagent kits. Fifty‐four melanomas (25 mucosal, 12 ocular, and 17 cutaneous) and 26 nevi (15 cutaneous and 11 mucosal) specimens were selected. The detection rate for one of the investigated oncoviruses was 17% in mucosal, 20% in ocular, and 0% in cutaneous melanomas (CMs). Despite the differences between groups seeming remarkable, there was no statistical significance (*p* > 0.5). Our data do not support a primary role of oncoviruses in melanoma carcinogenesis; however, the finding of HPV and EBV DNA in a considerable fraction of mucosal and OMs suggests that these viruses may act as cofactors in the development of extra‐CMs.

## 1 INTRODUCTION

Melanoma has been classified into subtypes based on the tissue from which the primary tumor arises. Cutaneous melanoma (CM), which arises in non‐glabrous skin, is the most common subtype; mucosal melanoma (MM), the rarest subtype, arises from melanocytes of the mucosal membranes; ocular melanoma (OM) develops from the melanocytes in the uveal tract of the eye.^1^

The etiology of melanoma is multifactorial, reflecting the interaction between host‐related and environmental risk factors.^1^ Host‐related factors consist of peculiar, genetically determined, phenotypic traits such as the light color of the skin, hair, and eye, scarce ability to tan, and propensity to burn, and high number of common and atypical nevi. Genetic susceptibility to melanoma is defined by the presence of germline mutations in high‐risk and high penetrance melanoma susceptibility genes, such as cyclin‐dependent kinase inhibitor 2A, present in 20%–45% of familial CM cases, and BRCA1‐associated protein‐1, found in up to 28% of families with CM and OM.^1^

With regard to environmental factors, the exposure to ultraviolet (UV) radiation is the main risk factor for CM.^1^ Nonsolar occupational risk factors for CM are: exposure to petroleum at automobile manufacturing plants, exposure to benzene and trichloroethylene in the clothing industry and biological/chemical workers and to pesticides in the agriculture industry, farmers and veterinarians.^1^

As for MM, since it mostly arises on surfaces which are not exposed to sun, this well‐known risk factor for CM is unlikely to be involved. Exposure to formaldehyde for sinonasal MM and cigarette smoking for oral MM have been suggested as risk factors.^2^

For OM, besides the host susceptibility factors, two professional occupations, welding, and cooking have been associated with tumor development for the exposure to cooking oil fumes, containing carcinogenic agents such as polycyclic aromatic hydrocarbons, and to intense artificial sources of optical radiation including UV, visible, and infrared light.^3^

The role of other environmental factors, such as the human oncoviruses, in the pathogenesis of melanoma has been poorly investigated. Oncoviruses include DNA and RNA viruses that usually are a necessary but not sufficient conditions for developing cancers.^4^

Only a few studies have focused on the involvement of human papillomaviruses (HPVs) in melanoma showing contradictory results.^5^, ^6^, ^7^, ^8^ The role of other oncoviruses, such as Epstein Barr virus (EBV) and human Herpesvirus 8 (HHV‐8), has never been studied.

The aim of this study was to investigate the association between oncoviruses and melanomas of the skin, mucosal membranes, and eye searching for the presence of high risk (HR)‐HPVs, EBV, and HHV‐8 DNA in melanoma tissue specimens. Archive materials of formalin‐fixed and paraffin‐embedded (FFPE) melanomas were examined using different polymerase chain reaction (PCR) reagent kits. The same tests have been performed in cutaneous and mucosal nevi, as control samples.

## 2 MATERIALS AND METHODS

A retrospective data search was conducted in the electronic archives of the Pathology Departments of three Hospitals: Galliera Hospital (Genoa) and University Hospitals of Turin and Cagliari.

FFPE tissue specimens of MMs and OMs diagnosed between January 1, 2000, and December 31, 2018, were selected. An analogous number of CMs and cutaneous and mucosal nevi diagnosed between January 1, 2018, and December 31, 2020, has been collected from the Galliera Hospital, Genoa.

From each FFPE block, two sections of 5 × 10 μm were cut, deparaffinized with xylene, and rehydrated through a graded series of distilled water‐ethanol solutions. QIAamp DNA FFPE Tissue Kit (QIAGEN) was used for DNA extraction. DNA was quantified with a NanoDrop 3300 fluorospectrometer using the RiboGreen dye diluted in TE buffer (Thermo Fisher Scientific).

One of the two section specimens obtained from each melanoma sample have been analyzed for the search of HPV DNA in the Pathology Department of the Galliera Hospital, according to the manufacturer's instruction. The other section specimens were sent to the Microbiology Laboratory of the University of Milano‐Bicocca, Milan, Italy, where the analysis for the search for other oncoviruses has been performed.

### 2.1 Virus detection

HPV detection and genotyping were performed using the Xpert HPV Assay. Xpert includes reagents for the simultaneous detection of 13 HR‐HPV types (HPV16, −18, −31, −33, −35, −39, −45, −51, −52, −56, −58, −59, and −68) and 1 possible HR‐HPV type (HPV66), a human reference gene (hydroxymethylbilane synthase), and an internal Probe Check Control (PCC). The 14 targeted HPV types are detected in five fluorescent channels: fluorescent channel 1 (HPV16), 2 (HPV18 and −45) (“HPV18/45”), 3 (HPV pool P3: HPV31, −33, −35, −52, and −58), 4 (HPV pool P4: HPV51 and HPV59) and 5 (HPV pool P5: HPV39, −56, −66, and −68). The human reference gene (fluorescent channel 6) verifies specimen adequacy. The PCC verifies reagent rehydration, PCR tube filling in the cartridge, probe integrity, and dye stability. In total, the assay utilizes six fluorescent channels for the detection of individual types of HPV, groups of HPVs, and the human reference gene. Each fluorescent channel has its own cutoff parameters for target detection/validity. If a sufficient signal is detected by the human reference gene (if the sample has sufficient cellularity), the assay results are reported as an overall “positive” if any type of targeted HPV is detected, or “negative.” Detailed technical descriptions of the assay have been reported elsewhere.^9^ The specimens with insufficient cellularity for any specific oncovirus analysis were identified as “not suitable” (Table 1).

**Table 1:** Clinical, histopathological and virological features of the melanoma cases

	Clinical−demographic features				Histological features				Virological investigations		
No	Melanoma type	Melanoma site	Sex	Age at diagnosis	Breslow thickness (mm)	Mitosis/mm2	Ulceration	Staging	HPV DNA	EBV DNA	HHV8 DNA
1	Mucosal	Vagina	F	60	Nr	Nr	Nr	Nr	Neg	NS	Neg
2	Mucosal	Conjunctiva	M	63	Nr	Nr	Nr	Nr	Neg	Neg	Neg
3	Mucosal	Small intestine	M	82	Nr	Nr	Nr	Metastasis	Neg	Neg	Neg
4	Mucosal	Large intestine	M	87	Nr	10/10 microscopic fields at 400x	Nr	Nr	Neg	Neg	Neg
5	Mucosal	Nasal cavity	M	82	Nr	Nr	Nr	Nr	Pos pool P5	Neg	Neg
6	Mucosal	Conjunctiva	M	47	1.3	2	Absent	pT1b	Neg	NS	Neg
7	Mucosal	Anal	F	40	3.7	3	present	pT3b	NS	NS	Neg
8	Mucosal	Nasal cavity	M	85	Nr	Nr	Nr	Nr	Neg	Neg	Neg
9	Mucosal	Oral commissure	M	62	Nr	Nr	Nr	Nr	Neg	Neg	Neg
10	Mucosal	Vulva	F	77	6.6	6	Present	pT4b	Neg	Neg	Neg
11	Mucosal	Lip	M	56	1.5	4	Absent	pT2a	Neg	Neg	Neg
12	Mucosal	Nasal cavity	M	48	Nr	0	Present	Nr	Neg	Neg	Neg
13	Mucosal	Anal	M	79	Nr	Nr	Nr	Recurrence at the site of the primary melanoma	Neg	Neg	Neg
14	Mucosal	Nasal cavity	M	87	Nr	20X10HPF	Nr	Nr	Neg	Neg	Neg
15	Mucosal	Vulva	F	83	5	3	Nr	pT4a	Neg	NS	Neg
16	Mucosal	Glans	M	70	1.1	1	absent	pT2a N0 sn	Neg	Neg	Neg
17	Mucosal	Glans	M	77	1.1	4	absent	pT2a	Neg	Neg	Neg
18	Mucosal	Vulva	F	76	Nr	Nr	Nr	Nr	Neg	NS	Neg
19	Mucosal	Vulva	F	73	0 (in situ)	0	Absent	pTis	Neg	Pos	Neg
20	Mucosal	Anal	F	60	Nr	Nr	Nr	Metastasis	Neg	Neg	Neg
21	Mucosal	Conjunctiva	M	45	Nr	Nr	Nr	Metastasis	Neg	NS	Neg
22	Mucosal	Conjunctiva	F	57	0 (in situ)	0	Absent	pTis	NS	Neg	Neg
23	Mucosal	Conjunctiva	M	54	5	Nr	present	Nr	Pos pool P5	Neg	Neg
24	Mucosal	Conjunctiva	M	78	1	Nr	Nr	Nr	Neg	Neg	Neg
25	Mucosal	Urethra	M	51	0 (in situ)	0	Absent	pTis	Neg	Neg	Neg
Total of positive specimens									2/23 (9%)	1/19 (5%)	0
26	Ocular	Choroid	M	50	Nr	Nr	Nr	Nr	Neg	NS	Neg
27	Ocular	Choroid	F	76	Nr	3x10HPF	Nr	pT2	NS	NS	Neg
28	Ocular	Choroid	M	56	Nr	Nr	Nr	pT3b	Neg	NS	Neg
29	Ocular	Iris	M	67	Nr	Nr	Nr	Nr	NS	NS	Neg
30	Ocular	Uvea	F	56	Nr	2	Nr	Nr	Neg	Neg	Neg
31	Ocular	Choroid	F	76	Nr	2	Nr	Nr	Pos pool P4	Pos	Neg
32	Ocular	Uvea	F	82	Nr	2	Nr	Nr	Neg	NS	Neg
33	Ocular	Choroid	M	77	Nr	2	Nr	Nr	Neg	Neg	Neg
34	Ocular	Uvea	M	71	Nr	3	Nr	Nr	Neg	Neg	Neg
35	Ocular	Uvea	F	49	1.4	Nr	Nr	Nr	Neg	NS	Neg
36	Ocular	Choroid	F	57	Nr	4	Nr	Nr	Neg	Neg	Neg
37	Ocular	Choroid	M	86	Nr	2	Nr	Nr	Neg	NS	Neg
Total of positive specimens									1/10 (10%)	1/5 (25%)	0
38	Cutaneous	Back	F	78	0.56	0	Absent	pT1a	NS	NS	Neg
39	Cutaneous	Back	M	74	0.84	0	Absent	pT1b	Neg	Neg	Neg
40	Cutaneous	Face	M	75	0.75	0	Absent	pT1a	Neg	Neg	Neg
41	Cutaneous	Lower limb	F	46	0.28	0	Absent	pT1a	NS	Neg	Neg
42	Cutaneous	Back	F	61	6.6	6	Present	pT4b	Neg	Neg	Neg
43	Cutaneous	Back	M	61	1.4	1	Absent	pT2a	Neg	Neg	Neg
44	Cutaneous	Face	M	77	1.67	1	Absent	pT2a	Neg	Neg	Neg
45	Cutaneous	Lower limb	F	78	2.25	6	Present	pT3b	NS	Neg	Neg
46	Cutaneous	Face	M	81	20	2	Present	pT4b	Neg	Neg	Neg
47	Cutaneous	Lower limb	F	63	1.01	1	Absent	pT2a	Neg	Neg	Neg
48	Cutaneous	Upper limb	F	52	1.89	2	Absent	pT2a	Neg	Neg	Neg
49	Cutaneous	Lower limb	M	54	1.02	3	Present	pT2b	Neg	Neg	Neg
50	Cutaneous	Upper limb	F	66	3.4	6	Absent	pT3a	Neg	NS	Neg
51	Cutaneous	Face	F	33	1.8	4	Present	pT2b	Neg	Neg	Neg
52	Cutaneous	Lower limb	F	82	7.3	4	Present	pT4b	Neg	Neg	Neg
53	Cutaneous	Lower limb	F	72	2.2	6	Present	pT3a	Neg	Neg	Neg
54	Cutaneous	Trunk	M	76	2.3	4	Absent	pT3a	Neg	Neg	Neg
Total of positive specimens									0	0	0

Abbreviations: EBV, Epstein Barr virus; HHV, human Herpesvirus; HPV, human papillomavirus; Neg, negative; Nr, not reported; NS, not suitable for analysis; Pos, positive.

Tissue sections were retrospectively analyzed by quantitative real‐time PCR assays also for DNA search of EBV (RealCycler EBAR‐UX/EBAR‐GX; Progenie Molecular) and HHV‐8 (Real Quality HHV‐8; AB Analitica), according to the manufacturer's instructions.

### 2.2 Statistical analysis

Fischer's exact test was used to analyze the differences in the virus detection rate between melanomas and nevi and between cutaneous and extra‐CMs. The results were considered statistically significant at *p* ≤ 0.05.

## 3 RESULTS

Overall, 54 melanoma specimens (25 MMs, 12 OMs, and 17 CMs) and 26 nevi were selected. Clinical, histopathological, and virological features of the studied specimens were described in Tables 1 and 2.

**Table 2:** Clinical, histopathological, and virological features of the studied nevi

Samples features								
	Clinical−demographic features				Histologic dysplasia	Virological investigations		
No	Nevus type	Nevus site	Sex	Age at diagnosis		HPV DNA	EBV DNA	HHV8 DNA
1	Cutaneous	Back	F	22	Yes	NS	Neg	Neg
2	Cutaneous	Back	F	29	No	Neg	Neg	Neg
3	Cutaneous	Back	F	29	No	Neg	Neg	Neg
4	Cutaneous	Back	F	29	Yes	Neg	Neg	Neg
5	Cutaneous	Back	F	29	No	Neg	Neg	Neg
6	Cutaneous	Face	M	57	No	Neg	Neg	Neg
7	Cutaneous	Back	F	38	Yes (mild)	Neg	Neg	Neg
8	Cutaneous	Back	M	55	Yes (mild)	Neg	Neg	Neg
9	Cutaneous	Lower limb	M	82	Yes (mild)	Neg	Neg	Neg
10	Cutaneous	Lower limb	M	40	Yes (mild)	Neg	Neg	Neg
11	Cutaneous	Lower limb	M	50	Yes (mild)	Neg	Neg	Neg
12	Cutaneous	Back	M	29	Yes (mild)	Neg	Neg	Neg
13	Cutaneous	Face	M	64	No	Neg	Neg	Neg
14	Cutaneous	Lower limb	F	78	No	Neg	Neg	Neg
15	Cutaneous	Back	F	78	No	Neg	Neg	Neg
Total of positive specimens						0	0	0
16	Mucosal	Conjunctiva	M	69	No	Neg	Neg	Neg
17	Mucosal	Conjunctiva	F	31	No	Neg	Neg	Neg
18	Mucosal	Conjunctiva	M	43	No	Neg	Neg	Neg
19	Mucosal	Conjunctiva	M	35	No	Neg	Neg	Neg
20	Mucosal	Conjunctiva	F	57	No	Neg	Neg	Neg
21	Mucosal	Conjunctiva	F	68	No	Neg	Neg	Neg
22	Mucosal	Conjunctiva	M	15	No	NS	Neg	Neg
23	Mucosal	Conjunctiva	M	30	No	NS	Neg	Neg
24	Mucosal	Conjunctiva	M	55	No	Neg	Neg	Neg
25	Mucosal	Conjunctiva	M	41	No	Neg	Neg	Neg
26	Mucosal	Conjunctiva	M	44	No	Neg	NS	Neg
Total of positive specimens						0	0	0

Abbreviations: EBV, Epstein Barr virus; HHV, human Herpesvirus; HPV, human papillomavirus; Neg, negative; NS, not suitable for analysis; Pos, positive.

**Table 3:** Summary of the the available literature on HPV detection in melanoma tissues

Authors	Year of publication	No of melanomas studied	Types of melanomas	Number of controls studied	Types of controls	Methods for HPV search and typing	No of HPV positive melanomas (%)	Type of HPVs in melanomas	No of the HPV positive controls (%)	Type of HPVs in controls
Scheurlen et al.	1986	36	Cutaneous melanomas	196	6 keratoacanthomas; 190 tumors of skin and other tissues	Hybridization with different HPV DNA probes	1 (3%)	HPV 38, 17	1 (0.5%)	HPV 37, 9
Astori et al.	1998	15	Not reported	20	Normal skin	PCR	2 (13%)	HPV 5, 20	7 (35%)	HPV GA1‐3, GA9‐4; GA3‐1
Takamiyagi et al.	1998	1	Cutaneous melanoma	0	_	rt‐PCR and ISH	1	HPV 16	_	_
Dreau et al.	1999	12	5 cutaneous melanomas; 7 lymph nodes melanoma tissues	Not reported	Normal skin	Immunoistaining	7 (58%)	HPV 16, 35	0	_
Henning et al.	1999	2	Cutaneous melanomas in patients with CIN III and breast cancers	Not reported	Tumors of the skin and other tissues	PCR and ISH	2	HPV 16	9	HPV 16
Miracco et al.	2001	54	53 cutaneous melanomas (45 acral lentigginous, 4 nodular, 4 lentigo maligna); 1 mucosal melanoma (superficial spreading conjunctival melanoma)	0	_	PCR	4 (7%)	HPV 16, 17, 18, 20, 24	_	_
Rohwedder et al.	2002	2	Mucosal melanomas	18	Normal skin	PCR	2	HPV 16 and putative novel HPV types (alb‐1, alb‐2, alb‐7, and alb‐10)	4 (22%)	HPV types 3, 54, and alb‐7
Roussaki‐Schulze et al.	2005	28	Cutaneous and mucosal melanomas	6	Melanocytic nevi, normal skin	PCR and RFLPA	5 (18%)	HPV 6, 16	0 (0%)	_
Dahlgren et al.	2005	35	Mucosal melanomas	0	_	PCR	2	HPV 16, 33	_	_
La Placa et al.	2005	51	Cutaneous melanomas	33 + 20	Dysplastic nevi, benign skin lesions, normal skin	GP‐PCR and MY‐PCR	14 (27%) with GP‐PCR; 11 (22%) with MY‐PCR	HPV 16, 18, 33, 35, 52, 58	8 dysplastic nevi (24%) with GP‐PCR and 5 dysplastic nevi (15%) with MY‐PCR; 0 benign lesions and normal skin	HPV 16, 18, 35
Ruer et al.	2009	100	Cutaneous melanoma	85	Healthy skin	Multiplex PCR + DNA microarray primary extension	33 (39%)	HPV 16, 5, 8, 12, 14, 21, 36, 22, 23, 38, 49, 76, 50	28 (42%)	HPV 11, 16, 42, 44, 57, 5, 8, 12, 21, 36, 47, 9, 22, 23, 38, 80, 76, 4, 65
Cun et al.	2013	melanoma cell lines	ocular melanoma	0	_	PCR	1	HPV 18	_	_
Our study	**2022**	**54**	**17 cutaneous, 25 mucosal, 12 ocular melanomas**	**26**	**15 cutaneous nevi; 11 conjunctival nevi**	**PCR**	**2 mucosal and 1 ocular melanomas (5%)**	**pool P5 (HPV39, −56, −66, −68) and pool P4 (HPV 51, −59)**	**0**	**_**

*Note*: The significance of bold value is to emphasize the results of the present work.

Abbreviations: HPV, human papillomavirus; ISH, In situ hybridization; PCR, polymerase chain reaction; rt‐PCR, reverse transcription polymerase chain reaction.

The HPV detection rate in all melanoma specimens (3/47 suitable cases, 6%) resulted higher than in nevi (0%) but the difference was not statistically significant (*p* = 0.54); likewise, the HPV detection rate in extra‐CMs (3/33 suitable cases, 9%) was higher than in CMs (0%) but the difference was not statistically significant (*p* = 0.54).

The EBV detection rate in all melanomas (2/39 suitable cases, 5%) resulted higher than in nevi (0%) but the difference was not statistically significant (*p* = 0.51); similarly, the EBV detection rate in extra‐CMs (2/24 suitable cases, 8%) was higher than in CMs (0%) but the difference was not statistically significant (*p* = 0.51).

Considering only the samples suitable for the investigation of both HPV and EBV, the detection rate of one of these oncoviruses was 17% in MMs (3/18 cases), 20% in OMs (1/5 cases), and 0% in CMs (0/13 cases). Despite the remarkable difference in the oncovirus detection rate between groups (in favor of MMs and OMs compared to CMs), these differences were not statistically significant (*p* > 0.5).

The genome of HHV‐8 has been detected neither in the specimens of melanomas nor in nevi. Detection of HPV and EBV in melanoma specimens was not associated with a particular age range nor with specific histological features (Table 1).

## 4 DISCUSSION

Oncogenic HPV types, mainly HPV16, are known to play a crucial role in the development of anogenital and oropharyngeal carcinomas.^4^ In addition, there is growing evidence that HPVs with cutaneous tropism may act as cocarcinogens with UV radiation in the development of nonmelanoma skin cancers.^10^ Moreover, cutaneous HPV infections may persist over several years on healthy skin.^11^ Only a few studies have examined the role of HPVs in cutaneous/mucosal/OM^5^, ^6^, ^7^, ^8^ and the role of other oncoviruses has been completely neglected.

The few papers investigating the involvement of HPV in CM and MM, including sporadic case reports^12^, ^13^ and a few case series,^5^, ^6^, ^7^, ^8^, ^14^, ^15^, ^16^, ^17^ found contradictory results. Regarding OM, only one study identified HPV18 infection in uveal melanoma cell lines suggesting that HPV might be involved in OM pathogenesis.^18^ Table 3 summarizes the available literature concerning HPV detection in melanoma tissues.

Our study is the first report of melanomas associated with “uncommon” HR‐HPV genotypes (pool P4 and P5); indeed, HPV16 and HPV18, the most common genotypes in all HPV‐related cancers worldwide,^4^ were also those most frequently detected types in melanomas in other studies (Table 3).

The HPV detection rate in our series (6%) is comparable to that found by Miracco et al. (7%).^16^ However, unlike several other studies (Table 3), we did not find HPV DNA in any of the CM cases but only in the extra‐cutaneous ones. The higher frequency of HPV detection in MMs (9%) compared to CMs (0%), although not statistically significant, suggests that HPV may play a role in the development of some of these cancers as well as those involving the anogenital mucosa.

The presence of HPV in mucosal and cutaneous tissues is not surprising given the mode of the virus transmission by direct contact but the detection of HPV in the choroid melanoma tissue (Table 1), never described in the literature, is noteworthy. Several recent studies have demonstrated the presence of HPV DNA in peripheral blood mononuclear cells, serum, and plasma from cervical cancer patients and healthy patients.^19^ Likewise, we can speculate that HPV infection, originating from the skin or mucosal membranes, has spread through the bloodstream reaching the melanocytes of the choroid.

The role of HPV in melanoma development could be either direct through infection of melanocytes or indirect, acting as a cofactor with UV radiation in sun‐exposed mucosal membranes (such as the conjunctiva). The oncogenic properties of the HR‐HPVs reside in the transforming activities of the viral oncoproteins E6 and E7 by interfering with cell cycle regulatory proteins and the immortalization of the infected cells.^4^

In our work, besides HR‐HPVs, the presence of EBV and HHV‐8 DNA has been investigated in melanomas and nevi specimens (Tables 1 and 2).

We detected EBV DNA in two extra‐CM specimens. Despite the difference in EBV detection rate between extracutaneous and CM specimens was not statistically significant, the EBV detection in a non negligible fraction of extra‐CMs (9%) may suggest a possible role of this virus in melanoma carcinogenesis. After the primary infection, EBV infection establishes a life‐long latency, predominantly in B cells, with occasional reactivation and shedding of progeny viruses. Since the lymphoid system is its “natural niche,” EBV is a risk factor for the development of B and T cell lymphoproliferative disorders.^20^ EBV oncogenesis is primarily driven by latent membrane protein 1 (LMP‐1), which acts as a constitutively activated CD40 receptor and is responsible for the transformation of B lymphocytes into proliferating lymphoblastoid cells. LMP 2A and EBV nuclear antigens also contribute to cancerogenesis by modulating key cellular processes.^20^ As it has been suggested to explain the presence and the role of EBV in breast cancer,^20^ we can assume that EBV‐positive lymphocytes may infiltrate the mucosal and ocular tissues and transmit EBV to the melanocytes.

Lastly, in our series, we reported the first case of melanoma that harbored both HR‐HPV and EBV DNA. This co‐infection has been described in other human cancers (cervical, breast, and prostate cancers),^20^ playing some role in carcinogenesis. Even if it is unclear which virus contributes to the first infection, the double infection may reinforce the capability of the viruses for cellular transformation.

In conclusion, the detection of oncoviruses DNA in a fraction of extra‐CMs (17% of MMs and 20% of OMs) does not indicate necessarily an association between these infections and tumors; however, it cannot be excluded that these viruses may act as cofactors, probably in association with other risk conditions, in the development of melanoma in uncommon body sites.

Notably, our work describes for the first time several new findings in the context of oncoviruses and melanoma: the detection of “uncommon” HR‐HPV genotypes in melanoma, differently from the frequently reported HPV16/18 genotypes; the presence of HPV‐EBV DNA in choroid melanoma tissue, supporting the hypothesis that these infections can spread from the skin/mucosal membranes via the bloodstream to other tissues and, finally, the presence of EBV DNA in MM.

Further studies are needed to better determine the role of HPV and EBV infection in melanoma, possibly by assessing their viral load to quantify virus activity.

## AUTHOR CONTRIBUTION

Giulia Ciccarese conceived the presented idea and wrote the manuscript with the support of Francesco Drago, Franco Rongioletti, and Carlo Tomasini. Davide Santinelli collected the data with support of Laura Pizzatti, Laura Atzori, and Luca Pilloni. Angela Pastorino, Francesco Broccolo, and Alice Urbani carried out the laboratory analysis. Aurora Parodi, Francesco Drago, and Franco Rongioletti helped supervise the project. all authors discussed the results and contributed to the final manuscript.

## CONFLICT OF INTEREST

The authors declare no conflict of interest.

## Data Availability

The data that support the findings of this study are available on request from the corresponding author. The data are not publicly available due to privacy or ethical restrictions.
